# The Context Matters: Longitudinal Effects of Cognitive Emotion Regulation Across Life Transitions in Men Experiencing Cancer Diagnosis, Retirement, and First-Time Fatherhood

**DOI:** 10.3390/bs16020193

**Published:** 2026-01-29

**Authors:** Mai Bjørnskov Mikkelsen, Hilde Randa, Mia Skytte O’Toole, Marlene Skovgaard Lyby, Mimi Yung Mehlsen

**Affiliations:** Department of Psychology and Behavioural Sciences, Aarhus University, 8000 Aarhus C, Denmarkmimim@psy.au.dk (M.Y.M.)

**Keywords:** emotion regulation, anxiety, depression, fatherhood, retirement, testicular cancer

## Abstract

**Objective:** To understand how emotion regulation may foster well-being through life transitions, one needs to consider situational factors and the specific strategy applied. Research has rarely investigated how emotion regulation relates to coping across diverse life transitions. Addressing this gap in the literature, the present paper investigated whether emotion regulation strategy use predicted distress during three distinct types of life transitions. **Methods:** A total of 305 men provided sociodemographic information and completed questionnaires assessing distress symptoms and cognitive emotion regulation strategy use monthly for a five-month period. Of the 305 men, 98 were first-time fathers, 34 had just received a cancer diagnosis, 81 were retiring, and 92 were control participants. **Results:** The results revealed that the prospective associations between emotion regulation strategy use and distress symptoms varied across life transitions. Self-blame was a predictor of increased anxiety in cancer patients (*p* = 0.012), acceptance was a predictor of increased depression in retirees (*p* = 0.007), rumination was a predictor of increased anxiety in fathers (*p* = 0.009), and for the control group, putting into perspective was associated with greater depression (*p* = 0.043), while catastrophizing were associated with greater anxiety (*p* = 0.003). **Conclusions:** The findings tentatively suggest that the adaptiveness of a given strategy varies depending on the specific life transition being experienced. This is consistent with the “person by situation by strategy” model, suggesting that successful emotion regulation is determined by the specific strategy applied, features of the situation, and person’s characteristics.

## 1. Introduction

There is a widespread consensus that emotions are functional and adaptive because they provide information that allow people to respond adaptively to their environment (Gendolla, 2014; Keltner & Gross, 1999). However, sometimes emotions need to be regulated in order for the individual to utilize them to achieve adaptive outcomes (Gross & Jazaieri, 2014). For example, you may need to upregulate anger to prepare for a competition or downregulate anger to avoid a conflict with your boss. Such attempts to change one’s emotional state are examples of emotion regulation, defined as “the processes by which individuals influence which emotions they have, when they have them, and how they experience and express them” (Gross, 1998, p. 275).

Emotion regulation is especially important during life transitions. Life transitions can be defined as periods of change—often following a major life event—that require adaption (Brammer, 1992; George, 1993). Perceptions of life transitions differ; whereas some are predominantly viewed as negative (e.g., losing a partner), others are predominantly viewed as positive (e.g., getting married), and yet again others are viewed as ambivalent or neutral (e.g., retirement, moving). However, common to life transitions is that they often involve changes in social roles, identity, worldview, and/or environmental demands (Brammer, 1992; George, 1993; Wheaton, 1990). These changes are often accompanied by emotional reactions that reflect the individual’s adaption to them (Brammer, 1992). Consequently, emotion regulation skills are a critical component in managing changes associated with life transitions (Brammer, 1992; Waizman et al., 2023). In support of this idea, research shows that emotion regulation is a predictor of adjustment to various life transitions such as the transition from high school to grad school (Finkelstein-Fox et al., 2018), the transition from working to retirement (Hed et al., 2024), and coping with bereavement (Eisma et al., 2023).

The importance of emotion regulation during life transitions highlights the need to consider whether emotion regulation functions as a generally adaptive process or varies depending on the specific demands of different types of transitions. Research suggests that there are individual and situational differences in which emotion regulation strategies people typically use and how effective people are at regulating their emotions (Doré et al., 2016; John & Gross, 2004; Kozubal et al., 2023; Matthews et al., 2021; Mikkelsen et al., 2023; O’Toole et al., 2021). In an attempt to provide an overarching model for such differences, Doré et al. (2016) introduced the “person by situation by strategy” model for successful emotion regulation (i.e., regulation that achieves the regulation goal). Following this model, successful emotion regulation is determined by person factors (e.g., gender, culture, psychological characteristics), situational factors (e.g., intensity and nature of the emotion), strategy factors (e.g., stage of emotion generation targeted, cognitive demands of the strategy), and their interaction. A number of studies have provided support for this model, including experimental studies (Kobylińska et al., 2023; Mikkelsen et al., 2021) and experience sampling studies (Hartmann et al., 2024; Livingstone & Isaacowitz, 2021; Springstein & English, 2023), emphasizing the importance of the context and person characteristics for emotion regulation success, as well as a recent meta-analysis of experience sampling studies finding that emotion regulation strategies are differentially associated with positive and negative affect in daily life (Boemo et al., 2022). Hence, to understand how emotion regulation may be a resource that promotes resilience and protects well-being through stressful life transitions, one needs to consider individual differences, situational factors, and the specific strategy applied. However, research has rarely investigated how emotion regulation relates to coping across multiple life transitions in samples with similar characteristics.

In the present study, we extend previous research by examining whether the use of emotion regulation strategies predicts distress symptoms across three types of life transitions: (1) becoming a parent for the first time, (2) receiving a cancer diagnosis, and (3) retiring. The life transitions were chosen to represent transitions predominantly perceived to be positive (i.e., first time parenthood), negative (i.e., receiving a cancer diagnosis) or neutral/ambivalent (i.e., retiring). The adjustment required during life transitions is often described as inherently cognitive, involving processes such as meaning-making and integration of change into one’s self-concept (Brammer, 1992). Accordingly, the present study focuses on cognitive emotion regulation strategies, which are aligned with the adaptive demands of life transitions.

## 2. Methods

The study was approved by the Danish National Committees on Health Research Ethics (ID = 1-10-72-159-16) and conducted in accordance with the Danish Data Protection Agency guidelines (ID = 2015-57-0098). The study was financially supported by a grant from the Danish Council for Independent Research (DFF—4180-00185).

### 2.1. Participants

Participants were recruited for a study exploring men’s psychological and physiological responses to life transitions. In the present study, only psychological data are reported. To be included, participants had to be above the age of 18 and proficient in the Danish language. Prior to inclusion, participants completed a screening interview assessing (a) anticipated major life events and (b) relevant life events occurring during the past year, as well as lifetime history of serious mental disorders. Participants were excluded if they reported a lifetime history of a serious mental disorder.

Group assignment was based on proximity to a single target transition. Participants were eligible for the first-time father or retirement groups if they expected to become a first-time father or to retire within the coming month. Participants were eligible for the cancer group if they were expected to be informed about their diagnosis within the next few days. To ensure mutually exclusive groups, participants were excluded from a given group if they reported having experienced any of the other target events during the past year (e.g., retirees were excluded if they had become a first-time father or received a cancer diagnosis within the past year). Control participants were eligible only if they did not expect to experience any of the target events within the coming month and had not experienced any of these events during the past year.

A total of 317 men agreed to participate. Of these, 305 men completed three or more assessments (out of six) and were included in our analyses. Our sample (age: M = 46.6, SD = 17.5, range: 20–78) represented the following four groups:

First-time fathers (*n* = 98, age: M = 30.1, SD = 4.3, range: 24–43 years), recruited from Aarhus midwifery practice at the planned consultation for their partners at 36 weeks pregnancy.

Testicular cancer patients (*n* = 34; age: M = 40.0, SD = 9.7, range: 20–57 years), recruited from Department of Oncology, Aarhus University Hospital. Patients were recruited immediately before being informed about their diagnosis.

Retiring men (*n* = 81, age: M = 65.4, SD = 3.3, range: 59–78 years) were recruited from advertisement in newspapers and local radio. The retiring men were included approximately one month before their planned date of retirement.

Controls (*n* = 92, age: M = 50.0, SD = 18.1, range: 20–74 years) were men who did not expect to experience (1) becoming fathers for the first time, (2) receiving a cancer diagnosis, or (3) retiring within the coming month and had not experienced any of these events during the 12-month-period before inclusion. Controls were recruited from advertisements in newspapers and through networks of participants who had already agreed to participate.

Participants reported the number of major life events they experienced during the observation period using a checklist of 20 events, e.g., becoming a father or grandfather, losing a child, starting or ending romantic relationships, starting or ending education or job, or experiencing serious accidents or disease. Fathers reported on average 1.8 events; cancer patients 1.3 events; retirees 0.9 events; controls 0.8 events.

### 2.2. Procedure

Participants received oral and written information about the project and provided written informed consent. Participants provided sociodemographic information at baseline and completed questionnaires assessing distress symptoms and emotion regulation strategy use at baseline and at one-, two-, three-, four-, and five-month follow-ups. All subjects were compensated with DKK 600 for completing the study.

## 3. Materials

### 3.1. Cognitive Emotion Regulation Strategies

Habitual cognitive emotion regulation strategy use were measured by the CERQ-short (Garnefski & Kraaij, 2006; Garnefski et al., 2001). The CERQ measures cognitive strategy use when faced with stressful life events (Garnefski & Kraaij, 2006). It yields scores for nine subscales each based on two items: self-blame (i.e., blaming oneself for experiences); acceptance (i.e., resigning oneself to what has happened); rumination (i.e., thinking about the feelings and thoughts associated with the event); positive refocusing (i.e., thinking about positive experiences instead of the actual event); planning (i.e., thinking about what steps to take and how to handle the event); positive reappraisal (i.e., thoughts of giving the event a positive meaning in terms of personal growth); putting into perspective (i.e., downgrading the importance of the event); catastrophizing (i.e., thoughts emphasizing the terror of what one has experienced), and blaming others (i.e., thoughts of putting the blame of what you have experienced on the environment or another person). High scores on the subscales indicate more frequent use of a strategy. For the overall CERQ-SF 18-item scale, Cronbach’s alpha was 0.81. For subscales, alpha-values ranged from 0.63 to 0.84, with only the self-blame subscale yielding an alpha-value below 0.73.

Via e-mail, participants were administered the CERQ-SF to measure emotion regulation strategies, on six occasions, each spaced one month apart. On each occasion, emotion regulation strategies were quantified by asking participants to indicate, in response to the prompt “When you have experienced negative events, how often…”, how frequently they used each of the 18 cognitive emotion regulation strategies, with response options ranging from almost never (1) to almost always (4).

### 3.2. Outcomes

Symptoms of anxiety and depression for the past four weeks were measured by the subscales for anxiety (four items) and depression (six items) of the Common Mental Disorder Questionnaire (CMDQ) (Christensen et al., 2005). High scores on the subscales indicate more symptoms of anxiety and depression, respectively. For the Common Mental Disorder Questionnaire 6-item depression-subscale, Cronbach’s alpha was 0.82, and for the 4-item anxiety subscale Cronbach’s alpha was 0.76. Participants received the CMDQ in the same email as the CERQ-SF.

## 4. Statistical Analyses

To assess whether there were changes in emotion regulation strategy use and distress symptoms over time, dependent *t*-tests were conducted comparing scores at T1 and T6. These tests were conducted to establish whether changes occurred in emotion regulation strategy use and distress symptoms over time, thereby providing the rationale for examining their longitudinal association.

To assess associations between emotion regulation strategy use and distress symptoms at the same time point, correlation analyses were conducted at T1. These analyses were conducted to assess whether emotion regulation strategy use and distress symptoms are related cross-sectionally which strengthens the rationale for examining the predictive value of emotion regulation strategy use on subsequent distress symptoms. Bivariate associations were examined using Pearson product–moment correlations. Formal tests of normality were not performed, as Pearson’s r is considered robust to moderate deviations from normality, particularly in samples of the present size. As a robustness check, supplementary Spearman rank-order correlations were conducted for the combined sample, yielding largely comparable results.

To assess the causal predictive effect of changes in emotion regulation strategy use on distress symptoms, time-lagged MLMs were conducted with emotion regulation use at time point_x_, controlled for time point_X−1_, and predicting distress at time point_X+1_, controlled for time point_x_. Thus, there was no overlap in time between the segments, potentially allowing for causal interpretations. With six assessment points, four segments were left to be included in the analyses. In these models, measurement points were nested in individuals and models were fitted with random intercepts. These models test whether emotion regulation strategy use is associated with subsequent changes in distress, strengthening inferences about potential directional or predictive effects.

All analyses were performed for the combined sample and separately for each group. Analyses were conducted in Stata version 14.

## 5. Results

A total of 305 participants filled in three or more of the monthly questionnaires and were included in our main analyses (response rate: 96.2%). Means and standard deviations for the nine emotion regulation strategies and two outcomes at first and last assessment are presented in Table 1. In the combined sample, significant differences in emotion regulation strategy usage were found for acceptance, rumination, refocus on planning, and positive reappraisal, with all strategies being used less frequently at T6 as compared to T1. Furthermore, significantly lower anxiety scores were reported at T6 as compared to T1. All significant changes, however, corresponded to either negligible or small effect sizes (Cohen’s d ≤ 0.3).

As shown in Table 1, subgroup analyses revealed distinct patterns. Among fathers, the strategies refocus-on-planning and positive reappraisal decreased modestly, while distress symptoms remained stable. Cancer patients displayed the largest reductions in strategy use and distress symptoms, with marked declines in positive reappraisal (d = 0.54) and anxiety (d = 0.48), and a medium-sized drop in rumination (d = 0.44). For retired participants, changes were minimal across all variables. Controls showed reductions in rumination, refocus on planning, positive reappraisal, and catastrophizing (d = 0.26–0.51), mirroring the overall pattern but with slightly stronger effects.

As shown in Table 2, self-blame, rumination, and catastrophizing correlated positively with both depression and anxiety across the combined sample at baseline, whereas strategies such as acceptance and positive reappraisal showed negligible or negative associations.

Subgroup analyses indicated notable variations. Among fathers, the negative associations between positive refocus and depression and between putting into perspective and depression were more pronounced than in the overall sample. Cancer patients showed strong links between self-blame, rumination, catastrophizing and emotional distress, and uniquely a negative correlation between positive reappraisal and anxiety. In retired participants, rumination, self-blame, and catastrophizing were related to both distress outcomes. Controls displayed a pattern similar to the total sample but with slightly stronger associations for catastrophizing and blaming others.

Overall, the correlation analyses verified concurrent associations between emotion-regulation strategies and distress measured at the same time points, though the direction and magnitude of associations varied across strategies and life-transition groups.

As shown in Table 3, few emotion-regulation strategies showed consistent time-lagged effects on later depression or anxiety. In the combined sample, higher self-blame predicted both increased depression (*z* = 2.38, *p* = 0.017) and anxiety (*z* = 2.32, *p* = 0.021), whereas positive reappraisal predicted lower depression (*z* = −2.68, *p* = 0.007).

Among fathers, no strategy predicted later depression, but rumination was a significant prospective predictor of higher anxiety (*z* = 2.60, *p* = 0.009). Cancer patients showed a similar pattern, with self-blame predicting increased anxiety (*z* = 2.51, *p* = 0.012). In retired participants, acceptance emerged as a positive predictor of subsequent depression (*z* = 2.69, *p* = 0.007). Among controls, putting into perspective predicted increased depression (*z* = 2.02, *p* = 0.043), while catastrophizing predicted increased anxiety (*z* = 2.93, *p* = 0.003).

## 6. Discussion

The present study revealed that the association between emotion regulation strategy use and distress symptoms varied across life transitions, although effect sizes were generally small. This finding suggests that emotion regulation strategies are not uniformly associated with adaptive outcomes, and that the adaptiveness of a given strategy may vary depending on the specific life transition being experienced. This interpretation should be considered tentative given the small effect sizes and the limited number of measurement intervals. Additional research is needed to verify the effects. Nonetheless, the observed pattern is consistent with the “person by situation by strategy” model, suggesting that successful emotion regulation is determined by the specific strategy applied, the features of the situation, and person’s characteristics (Doré et al., 2016). This finding also provides preliminary support for the related perspective that no emotion regulation strategy is in itself inherently adaptive or maladaptive (Aldao et al., 2015; Bonanno & Burton, 2013; Sanchez-Lopez, 2021). Rather, it is the ability to align strategy use with the context and one’s personal resources (i.e., emotion regulation flexibility) that is proposed to be adaptive (Aldao et al., 2015; Bonanno & Burton, 2013; Kobylińska & Kusev, 2019; Sanchez-Lopez, 2021; Springstein & English, 2024).

Concerning the association between specific strategies and distress symptoms across the four time-lagged intervals, we found that self-blame was a predictor of greater depression and anxiety, and reappraisal was a predictor of less depression across participants, disregarding groups. However, in support of variation in adaptiveness of strategies across individuals and situations, the only significant result for self-blame and reappraisal at the group level was that self-blame was a predictor of anxiety in cancer patients. This finding is consistent with systematic reviews finding that self-blame is associated with increased distress following a diagnosis of a chronic physical health condition (Callebaut et al., 2017) and in patients with a physical health condition (Jannati et al., 2020). For patients with health conditions, self-blame may elicit anxiety because it fosters a sense of personal responsibility for an uncertain disease and its progression (Callebaut et al., 2017). Clarifying the relationship between self-blame and an exaggerated sense of personal responsibility for one’s illness may help elucidate the mechanisms linking self-blame to anxiety.

Turning to the other strategies at group level, acceptance was associated with greater depression for the retired group. In the present study, acceptance indexes resignation to one’s circumstances, suggesting a more passive stance characterized by yielding to one’s circumstances. In the case of retirement, a flexible, active stance may be particularly beneficial as retiring can be considered a dynamic process in which the retiree must adjust to a loss of agency and identity in the work sphere and seek out other domains in which agency, meaning, and purpose can be expressed (e.g., volunteer work, leisure activities, social engagements) (Topa & Valero, 2017; Wang et al., 2011; Zhan et al., 2025).

For new fathers, rumination (i.e., thinking about feelings and thoughts associated with stressful events) was associated with greater anxiety. This aligns with broader evidence identifying rumination and other forms of disruptions of inner speech as transdiagnostic factors linked to anxiety across multiple populations (Daho & Monzani, 2025; McLaughlin & Nolen-Hoeksema, 2011; Moulds & Mcevoy, 2025). Compared to cancer patients and retirees, new fathers may experience greater perceived agency and expectations in their life transition. From the perspective of control theories of self-regulation, rumination may arise in the context of stressful life transitions because of discrepancies between expected rates of progress towards a goal and actual progress (Moulds & Mcevoy, 2025). When the discrepancy is resolved, the rumination will stop (Moulds & Mcevoy, 2025; Watkins, 2008). In the context of new fatherhood, discrepancies may arise when one’s experience of parenting falls short of internal or societal ideals of effective parenting. As such ideals may be difficult to attain, the discrepancies may persist, leading to rumination which may contribute to heightened anxiety.

For the control group, putting into perspective (i.e., minimizing the importance of an event) was associated with greater depression and catastrophizing (i.e., maximizing the terror of an event) was associated with greater anxiety. Concerning catastrophizing, the findings align with findings from a recent meta-analyses of 53 studies linking catastrophizing to state and trait anxiety (Yao et al., 2023). However, findings from previous studies examining putting into perspective in healthy populations have been mixed: some report a positive association with depression (Guo & Pan, 2025), others a negative association (Kökönyei et al., 2024; Martin & Dahlen, 2005), and still others find no significant relationship (Garnefski & Kraaij, 2018; Stikkelbroek et al., 2016). In the present study, reported emotion regulation strategy use by individuals who did not undergo life transitions likely reflects regulation in response to daily hassles. Speaking to the importance of calibrating emotion regulation strategies to the context, minimizing or exaggerating the significance of daily life events and their consequences may contribute to distress because they create a mismatch between the emotional response and situational demands (Kobylińska & Kusev, 2019; Springstein & English, 2024). Taken together, the findings of the present study tentatively suggest that the context may play a crucial role in determining when and for whom particular emotion regulation strategies support or hinder emotional functioning.

Concerning general changes in strategy use over time, the results indicated that the control group experienced more changes than the other groups. Participants in this group were selected because they did not expect to experience any major life events during the observation period, yet changes in strategy use nevertheless occurred over time. Notably, these shifts were observed while their overall level of psychological well-being remained stable. This finding suggests that strategy use varies over time and cannot be assumed to remain stable over a five-month period in a community sample. Whether this reflects a methodological artefact—such as altered reporting styles due to repeated assessments—or genuine natural fluctuations related to events in their everyday lives cannot be determined based on the present data. However, variability in strategy use over extended periods of time have been reported in previous studies with healthy samples suggesting that it is likely normative rather than a methodological artefact (Benson et al., 2019; Blanke et al., 2020; Elkjaer et al., 2022; Mikkelsen et al., 2023). It is plausible that the life transition groups varied their emotion regulation strategies less because the most salient challenges they faced were relatively stable and grouped in a single life domain. By contrast, the most salient stressors for the control group likely varied over time. This interpretation should be viewed as tentative; however it is consistent with the notion that adaptive emotion regulation involves matching strategies to contextual demands and person-level characteristics (Aldao et al., 2015; Bonanno & Burton, 2013; Springstein & English, 2024). In sum, stable, unchanging reports of strategy use may represent a more unusual pattern than naturally occurring variation in the application of emotion-regulation strategies.

### 6.1. Implications and Future Directions

The findings of this study are preliminary, but if confirmed by future research, they have several important implications for theory and practice. First, the findings provide support for theoretical frameworks emphasizing the role of person-, situational- and regulation-related factors for successful emotion regulation strategy use (e.g., the “person by situation by regulation” model, Doré et al., 2016). Specifically, the results suggest that successful emotion regulation strategy use may vary dependent on the type of life transition. These frameworks can be used to systematically investigate the contributions of person factors, situational factors, and regulation factors to successful emotion regulation. By selectively manipulating variables of interest and holding other variables constant, researchers can more precisely identify sources of emotion regulation success. For example, comparing different strategies for similar populations going through the same type of life transitions, or examining different populations exposed to the same type of life transitions and using the same strategies. Although a growing body of research have examined components of the person by situation by strategy model and similar models (e.g., (Bonanno & Burton, 2013; Goubet & Chrysikou, 2019; Pruessner & Ortner, 2025), such investigations have rarely focused on life transitions. Taking this approach acknowledges that successful emotion regulation arises from the interplay of person-, situation-, and regulation-related factors, and provides a clear framework for isolating and testing the contribution of each factor during life transitions.

Turning to implications for practice, the findings indicate that interventions aimed at fostering well-being during life transitions may benefit from a tailored and individual approach to promoting successful emotion regulation. This recommendation is consistent with findings from a recent meta-analysis demonstrating that personalized psychological interventions are associated with superior outcomes compared to standardized psychological interventions (Nye et al., 2023). One step in this direction could be to map and contextualize emotion regulation strategy use and success (Aldao et al., 2015) For example, having a person describe emotion regulation attempts, strategies used, their effectiveness in achieving the individual’s goals and the context. This information may then be used by practitioners to obtain a better understanding of individual precursors of successful versus unsuccessful regulation and to help the person increase flexible and contextually appropriate strategy use (Aldao et al., 2015). These ideas resonate with recent suggestions for therapeutic interventions aimed at promoting flexible and context sensitive emotion regulation (Sharma & Singh, 2025; Veilleux et al., 2022). For example, the therapeutic concept of a “thinking threshold” helps clients identify when the intensity of their emotions compromises cognitive functioning, signaling the need to shift from cognitively taxing emotion regulation strategies (e.g., reappraisal) to less cognitively taxing strategies (e.g., exercise or progressive muscle relaxation; Veilleux et al., 2022). Such interventions promoting flexible emotion regulation may be particularly important in the context of life transitions which involve periods of substantial changes and emotional reactions to these changes (Brammer, 1992).

### 6.2. Limitations

The present findings should be interpreted in light of several limitations. First, research suggests that person characteristics such as sex and age may influence emotion regulation (Doré et al., 2016; Kelly et al., 2008; Mikkelsen & O’Toole, 2022; Mikkelsen et al., 2023). Only male participants were included in the present study because the original study protocol involved the collection of biological markers that could be affected by sex-specific factors. Hence, it is unclear whether the results generalize beyond men. Furthermore, age may represent a potential confounding factor in the present study as the new fathers were on average younger than the other groups, the retirees were older, and the cancer patients encompassed a wider age range. Consequently, it remains unclear whether the findings are attributable to age differences or to the nature of the life transitions themselves. In addition to age, the recruitment context differed across groups, and baseline psychological burden may also have varied between them. However, age, recruitment context, and level of psychological burden are inherently linked to the life transitions studied (e.g., retirees are, by definition, older), and adjusting for these factors could blur transition-specific patterns. By leaving these factors unadjusted, we were able to examine how the associations between emotion regulation strategies and distress vary across transitions as they naturally occur. In order to disentangle effects of factors such as age, sex, psychological burden, and recruitment context from those of life transitions, future studies may compare positive, negative, and neutral life transitions that occur at comparable ages, for all sexes, involve similar recruitment contexts and similar levels of psychological burden at baseline.

Second, in the present study we assessed self-reported habitual emotion regulation and self-reported distress every month over five months, resulting in four intervals for the time-lagged analyses. It is plausible that a more fine-grained (e.g., daily or momentary level) or a multi-method assessment strategy may have revealed different results. Especially considering the well-known problems with common method variance (Podsakoff et al., 2003) and considering preliminary evidence that self-reported habitual emotion regulation may correlate only moderately, or less than moderately, with daily reports of emotion regulation strategy use (Koval et al., 2023). Future research could mitigate such concerns by incorporating multiple methods (e.g., experience sampling or ambulatory assessments) and multiple data sources, such as pairing self-reported strategy use with more objective indicators of distress (e.g., physiological measures or clinician-rated symptoms; Yasin et al., 2023). Such approaches would reduce the risk of common method variance influencing the results, allow researchers to capture moment-to-moment variability in response to the contextual demands of each transition, and are needed to validate and extend the present findings. Additional and more recent approaches to studying emotion regulation may also prove valuable. For example, the emerging notion of polyregulation (i.e., using several strategies simultaneously) may offer additional insight into the associations between emotion regulation strategy use and distress symptoms during life transitions (Hartmann et al., 2024; Ladis et al., 2023). 

Third, the life transitions were captured at different time points. Fathers and retirees were assessed from approximately one month before the transition to four months after the transition, while cancer patients were assessed from the day, they received their diagnosis to five months after. This difference in timing may have impacted the results as the new fathers and the retirees had not yet fully transitioned to their new roles during the first month of assessment, whereas the cancer patients had. However, the inclusion of cancer patients adds substantial value to the study, as it broadens the understanding of emotion regulation across qualitatively different life transitions.

Fourth, although participants were recruited based on whether they had experienced one of the major life transitions of interest (i.e., retirement, becoming a father, receiving a cancer diagnosis), and the control group was selected specifically because they had not undergone any of these life transitions, the control group still reported some life events during the observation period (mean *N* = 0.8). While the number of life events were numerically lower than in all other groups, it still represents a potential limitation, as these events may have affected the results for the control group. Future research may benefit from restricting the control group to participants who report no life events during the observation period to provide a more robust comparison to participants undergoing life transitions. On the other hand, events that occurred in the present control group served to account for the impact of similar events in the transition groups that were not related to the focal transitional events under investigation.

Fifth, engagement in psychological support or treatment during the study period was not assessed and may have influenced changes in emotion regulation and distress. This constitutes a potential source of unmeasured confounding typical of longitudinal, naturalistic designs. Future studies would benefit from explicitly assessing concurrent psychological support or treatment to better disentangle the effects of life transitions from intervention-related influences.

## 7. Conclusions

The present study investigated the prospective association between cognitive emotion regulation strategy use and distress symptoms during three distinct life transitions: becoming a father for the first time, receiving a cancer diagnosis, and retiring. The results revealed variation in associations across life transitions suggesting that the adaptiveness of a given strategy may depend on the specific life transition being experienced.

## Figures and Tables

**Table 1:** Changes in strategies and outcomes from T1 to T6 in all transition groups.

	T1	T6	t	*p*	Cohen’s d
All (*n* = 300)	M (SD)	M (SD)			
Self blame	4.1 (1.7)	4.1 (1.7)	0.48	0.631	0.03
Acceptance	7.3 (1.9)	7.0 (1.9)	2.53	0.012	0.15
Positive refocus	5.3 (1.8)	5.5 (2.0)	−1.85	0.065	0.11
Rumination	6.3 (1.9)	5.7 (1.9)	5.10	<0.001	0.30
Refocus on planning	7.9 (1.6)	7.5 (1.7)	3.74	<0.001	0.22
Positive reappraisal	7.0 (1.8)	6.5 (1.9)	5.28	<0.001	0.31
Catastrophizing	3.7 (1.5)	3.5 (1.4)	1.86	0.064	0.11
Blame others	4.7 (1.7)	4.8 (1.6)	−1.78	0.076	0.10
Putting into perspective	6.6 (1.8)	6.7 (2.01)	−0.37	0.710	0.02
Symptoms depression	2.1 (2.5)	2.2 (2.8)	−0.80	0.424	0.05
Symptoms anxiety	2.0 (2.1)	1.6 (1.7)	3.41	<0.001	0.20
Fathers (*n* = 97)					
Self blame	4.4 (1.7)	4.2 (1.8)	0.72	0.476	0.07
Acceptance	7.5 (1.7)	7.4 (1.8)	1.03	0.308	0.10
Positive refocus	5.2 (1.9)	5.4 (2.1)	−1.20	0.233	−0.12
Rumination	6.1 (1.9)	5.9 (1.9)	1.29	0.199	0.13
Refocus on planning	8.1 (1.5)	7.8 (1.7)	2.08	0.041	0.21
Positive reappraisal	7.0 (1.9)	6.5 (1.9)	2.63	0.010	0.27
Catastrophizing	3.7 (1.6)	3.8 (1.6)	−0.84	0.403	−0.09
Blame others	5.0 (1.6)	5.2 (1.7)	−1.03	0.308	−0.10
Putting into perspective	6.6 (2.0)	6.8 (2.1)	−0.97	0.336	−0.10
Symptoms depression	2.2 (2.6)	2.5 (2.9)	−1.37	0.175	−0.14
Symptoms anxiety	2.3 (2.2)	2.0 (1.7)	1.72	0.088	0.18
Cancer patients (*n* = 33)					
Self blame	4.3 (1.8)	4.4 (1.8)	−0.51	0.616	−0.09
Acceptance	7.8 (2.0)	7.2 (1.5)	1.79	0.084	0.31
Positive refocus	5.3 (1.7)	5.0 (1.9)	1.08	0.288	0.19
Rumination	6.5 (2.0)	5.6 (1.7)	2.52	0.017	0.44
Refocus on planning	7.5 (1.7)	7.0 (1.8)	1.46	0.154	0.25
Positive reappraisal	7.2 (1.9)	6.2 (1.8)	3.08	0.004	0.54
Catastrophizing	3.7 (1.2)	3.4 (1.2)	0.98	0.332	0.17
Blame others	3.4 (1.7)	4.1 (1.9)	−2.03	0.051	−0.35
Putting into perspective	6.8 (1.9)	6.5 (1.8)	0.89	0.381	0.16
Symptoms depression	3.8 (3.0)	3.7 (3.8)	0.18	0.859	0.03
Symptoms anxiety	3.5 (2.7)	2.5 (2.7)	2.73	0.010	0.48
Retired (*n* = 79)					
Self blame	3.8 (1.4)	3.8 (1.7)	0.00	1.000	0.00
Acceptance	7.0 (2.0)	6.8 (2.2)	1.02	0.309	0.12
Positive refocus	5.5 (1.8)	5.6 (2.0)	−0.75	0.455	−0.08
Rumination	6.1 (1.8)	5.7 (2.1)	1.85	0.068	0.21
Refocus on planning	7.6 (1.7)	7.3 (1.4)	1.36	0.178	0.15
Positive reappraisal	6.6 (1.7)	6.5 (1.8)	0.54	0.589	0.06
Catastrophizing	3.6 (1.3)	3.5 (1.4)	1.00	0.320	0.11
Blame others	4.8 (1.6)	4.9 (1.6)	−0.66	0.511	−0.07
Putting into perspective	6.4 (1.8)	6.6 (2.2)	−1.04	0.303	−0.12
Symptoms depression	1.8 (2.2)	2.0 (2.8)	−0.79	0.434	−0.09
Symptoms anxiety	1.5 (1.6)	1.3 (1.6)	1.38	0.171	0.16
Controls (*n* = 91)					
Self blame	4.1 (1.7)	4.0 (1.8)	0.46	0.650	0.05
Acceptance	7.1 (1.9)	6.8 (2.0)	1.41	0.163	0.15
Positive refocus	5.2 (1.8)	5.7 (2.1)	−1.83	0.070	−0.19
Rumination	6.5 (2.0)	5.6 (1.8)	4.61	<0.001	0.48
Refocus on planning	8.0 (1.4)	7.6 (1.9)	2.51	0.014	0.26
Positive reappraisal	7.4 (1.6)	6.6 (1.9)	4.87	<0.001	0.51
Catastrophizing	3.7 (1.8)	3.3 (1.3)	2.92	0.004	0.31
Blame others	4.7 (1.5)	4.7 (1.4)	−0.18	0.855	−0.02
Putting into perspective	6.8 (1.7)	6.7 (2.1)	0.66	0.514	0.07
Symptoms depression	1.6 (2.1)	1.5 (2.1)	0.67	0.507	0.07
Symptoms anxiety	1.4 (1.7)	1.2 (1.3)	1.30	0.197	0.14

Note. T1 = first assessment, T6 = last assessment, *t* = paired samples *t*-test. Strategy score range 2–10; depression score range 0–24; anxiety score range 0–16.

**Table 2:** Concurrent (T1) correlations between emotion regulation strategies and distress across life-transition groups.

Emotion-Regulation Strategy	All		Fathers		Cancer Patients		Retirees		Controls	
	Depression	Anxiety	Depression	Anxiety	Depression	Anxiety	Depression	Anxiety	Depression	Anxiety
Self-blame	0.32 **	0.38 **	0.34 **	0.40 **	0.29	0.47 **	0.37 **	0.24 *	0.31 **	0.37 **
Acceptance	0.05	0.02	0.03	−0.10	−0.08	−0.12	0.15	0.10	−0.03	−0.01
Positive refocus	−0.11	0.02	−0.21 *	−0.09	0.01	0.18	−0.09	0.09	−0.05	0.00
Rumination	0.19 **	0.25 **	0.04	0.14	0.28	0.36 *	0.28 *	0.38 **	0.24 *	0.22 *
Refocus on planning	−0.06	−0.06	0.01	−0.06	−0.04	−0.01	−0.03	−0.10	−0.12	−0.07
Positive reappraisal	0.00	−0.07	0.09	−0.01	−0.29	−0.36 *	0.09	0.08	−0.05	−0.14
Putting into perspective	−0.10	−0.12 *	−0.21 *	−0.18	−0.24	−0.17	0.13	0.06	−0.18	−0.30 **
Catastrophizing	0.27 **	0.29 **	0.16	0.24 *	0.34 *	0.22	0.33 **	0.40 **	0.41 **	0.45 **
Blame others	−0.02	−0.02	0.00	−0.10	−0.14	−0.05	0.10	0.23 *	0.21 *	0.23 *

Note. Pearson correlation coefficients are shown. Correlations reflect concurrent associations between emotion-regulation strategies and distress (depression and anxiety) measured at the same time points. Results are presented separately for each life-transition group. *p* < 0.05 (*), *p* < 0.01 (**), two-tailed.

**Table 3:** Results from time-lagged multilevel analyses assessing prospective associations between strategy use and distress symptoms.

*Group*/Outcome	Self-Blame		Accept.		Positive Refocus		Rumination		Planning		Reappraisal		Perspective		Catastrophizing		Blame Others	
	*z*	*p*	*z*	*p*	*z*	*p*	*z*	*p*	*z*	*p*	*z*	*p*	*z*	*p*	*z*	*p*	*z*	*p*
All Depression	**2.38**	**0.017**	1.25	0.210	0.16	0.874	−0.78	0.435	0.17	0.867	**−2.68**	**0.007**	0.30	0.763	−0.49	0.621	0.49	0.626
All Anxiety	**2.32**	**0.021**	1.12	0.265	−0.52	0.604	0.59	0.555	1.83	0.068	−1.57	0.116	1.04	0.298	1.07	0.285	1.36	0.175
Fathers Depression	1.61	0.107	−0.67	0.504	−0.13	0.897	1.13	0.257	1.75	0.080	−1.29	0.196	−1.36	0.173	0.30	0.766	0.83	0.408
Fathers Anxiety	0.15	0.879	−0.04	0.965	−0.48	0.631	**2.60**	**0.009**	1.75	0.081	−1.51	0.132	−0.32	0.746	1.20	0.229	0.11	0.914
Cancer Depression	0.45	0.654	0.44	0.660	0.22	0.825	−1.47	0.143	0.85	0.395	−1.76	0.078	0.17	0.869	−1.93	0.054	−0.29	0.770
Cancer Anxiety	**2.51**	**0.012**	1.79	0.074	−0.26	0.792	0.01	0.995	0.40	0.692	−0.45	0.651	0.19	0.852	−0.51	0.610	1.34	0.180
Retirees Depression	0.40	0.692	**2.69**	**0.007**	0.50	0.618	−0.25	0.799	−1.44	0.149	−1.56	0.119	−0.17	0.867	−1.04	0.300	−0.37	0.714
Retirees Anxiety	0.42	0.678	1.41	0.159	0.19	0.851	1.35	0.178	0.25	0.802	0.12	0.901	1.25	0.213	−0.52	0.603	1.80	0.071
Controls Depression	1.71	0.087	0.56	0.577	−0.05	0.963	−1.06	0.289	−0.74	0.462	−0.98	0.326	**2.02**	**0.043**	1.69	0.091	0.89	0.373
Controls Anxiety	1.65	0.098	1.64	0.101	−0.03	0.977	−0.66	0.507	−0.05	0.963	0.76	0.445	1.38	0.169	**2.93**	**0.003**	−0.27	0.784

Note. *z* = standardized test statistic; *p* = two-tailed significance. Significant results (*p* < 0.05) are shown in bold.

## Data Availability

Dataset available on request from the authors—The raw data supporting the conclusions of this article will be made available by the authors on request.
